# The gut microbiome of healthy long-living people

**DOI:** 10.18632/aging.101771

**Published:** 2019-01-15

**Authors:** Feilong Deng, Ying Li, Jiangchao Zhao

**Affiliations:** 1 Department of Animal Science, Division of Agriculture, University of Arkansas, Fayetteville, AR 72701, USA; 2 Farm Animal Genetic Resources Exploration and Innovation Key Laboratory of Sichuan Province, Sichuan Agricultural University, Chengdu, Sichuan, China

**Keywords:** gut microbiome, healthy aging, diversity, probiotics, SCFAs

The number of the elderly (>65 years old) is projected to reach 1.6 billion by 2050 worldwide, which poses substantial challenges to the economy, the society and the health care system. Thus, new solutions are imperative to mitigate age-related health problems. The human gut harbors trillions of bacteria (known as the gut microbiota), which play important roles in health and diseases. Several recent studies have characterized the human gut microbiome in the elderly. Gut microbial diversity generally decreases when people age [1], which is likely due to changes in physiology, diet, medication, and lifestyles. Decreased diversity, considered an indicator of an unhealthy microbiome, has been linked to different chronic conditions such as obesity and type 2 diabetes. In addition to decreased diversity, the changes of the gut microbiome composition to an imbalanced state, i.e. dysbiosis, also correlates with frailty, inflammation, and neurodegenerative disorders such as Alzheimer’s disease and Parkinson’s disease (PD) in the elderly [2]. In fact, the causality of the gut microbiome in PD has even been established in a mouse model, revealing the significance of the gut microbiota in causing motor deficits and microglia activation [3].

Given the fact that most of the elderly experience gut associated comorbidities, it is extremely challenging to define a healthy gut microbiome in this population. Changes in the gut environment such as inflammation, leaky gut, production of reactive oxygen species and application of medications can all affect the gut microbiome. In that regard, centenarians have been used as a model of healthy aging because of their capability to delay or avoid chronic diseases [4]. Therefore, the gut microbiome in this cohort might be used to define a healthy gut microbiome. The genetics, and recently epigenetics, of the centenarians have been extensively investigated, but relatively little is known about their gut microbiotas until now.

Kong and colleagues examined the gut microbiome of a cohort of healthy, long-living Chinese individuals including nonagenarians (90-99 years old) and centenarians (≥100 years old) in Dujiangyan, Sichuan, China. They found that this cohort of long-living people possesses a more diverse gut microbiota than younger adults, contradictory to conventional views. They also found that a group of bacteria, members of which are known short-chain fatty acid (SCFA) producers such as *Clostridium* cluster XIVa, are enriched in the long-living Chinese [5].

To verify their discovery, they analyzed an independent data set from a cohort of an Italian group. Consistently, the long-living Italians also had more diverse gut microbiotas than the younger group. When they combined the Italian and the Chinese data sets, they found that although the gut microbiota structures are significantly different, probably due to the differences in diet, genetics and environment, 11 of the top 50 bacterial features that differentiate the long-living individuals from the younger group were shared. Again, these features included the greater microbiome diversity and several enriched OTUs (operational taxonomic units) related to SCFA production [5]. In a follow-up study, Kong and colleagues showed that the greater gut microbiome diversity in the long-living people was also observed in two more independent cohorts: one from Jiangsu, China and the other from Japan [6].

**Figure 1:**
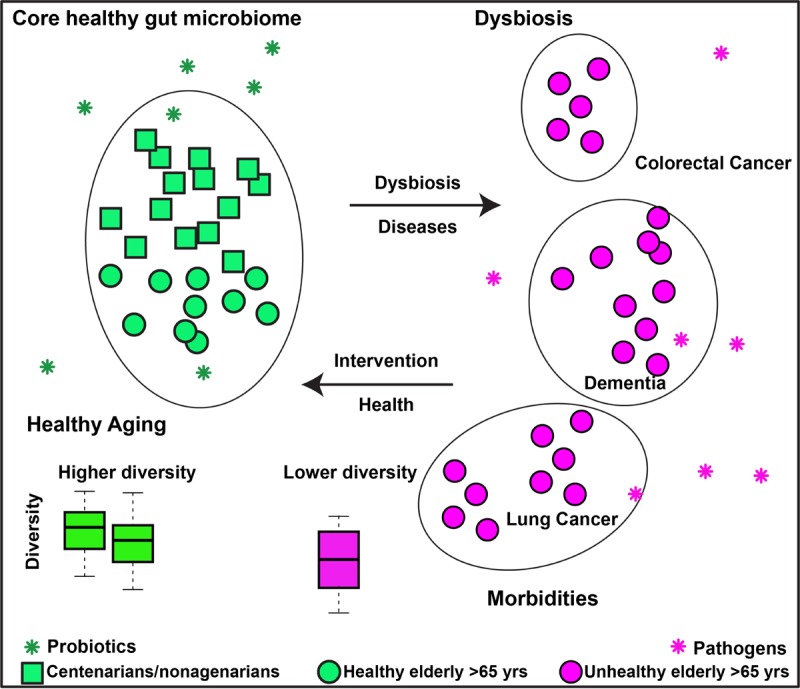
**Working hypothesis of gut microbiome and healthy aging.** We hypothesize that a diverse and balanced gut microbiome enriched with probiotics contributes to healthy aging; Modulation of the imbalanced gut microbiome in the elderly with morbidities would alleviate the symptoms and increase the quality of their lives.

These studies clearly revealed that more diverse and balanced gut microbiotas are present in healthy, long-living people, whereas disturbed gut microbiotas with dysbiosis are observed in the elderly who suffer from different comorbidities. We thus hypothesize that modulation of the gut microbiome (e.g., via diet, probiotics) to maintain a healthy gut microbiome will promote healthy aging. We further hypothesize that modulation of the disturbed gut microbiome to a healthy one in the elderly with chronic diseases will alleviate their symptoms and increase the quality of their lives (Figure 1).

One rationale behind this hypothesis is inflammaging, i.e. increased chronic, low-grade inflammation in the elderly, which is associated with different chronic diseases. SCFAs are important in maintaining gut hemostasis. SCFAs provide the primary energy for colon epithelial cells and possess anti-inflammation properties. The enrichment of these SCFA producers in long-living individuals suggests that these bacteria might reduce inflammation and its resulting damage in this cohort, which likely contributed to their healthy aging.

Of note, most of these studies are observational and based on association type of analysis. One key question in the gut microbiome field is the establishment of causality. A recent study using the short-lived African turquoise killifish as a model shows the effect of the gut microbiome in aging-related measures, which is a promising step towards the establishment of causality [7]. Further studies such as multi-omics approaches, fecal matter transplant from long-living people, the elderly, and young adults to animal models, and intervention-based clinical trials are warranted to pinpoint the functions of the gut microbiome in healthy aging and to identify novel approaches to modulate it for healthy aging.
